# Association of left ventricular geometry with outcomes and treatment response in atrial fibrillation and heart failure with preserved ejection fraction

**DOI:** 10.3389/fmed.2026.1824509

**Published:** 2026-05-04

**Authors:** Lan Ren, Xiaodong Peng, Mingxiao Li, Liu He, Ning Zhou, Xiaoxia Liu, Song Zuo, Jianzeng Dong, Changsheng Ma, Xueyuan Guo

**Affiliations:** Department of Cardiology, Beijing Anzhen Hospital, Capital Medical University and National Clinical Research Center for Cardiovascular Diseases, Beijing, China

**Keywords:** atrial fibrillation, heart failure with preserved ejection fraction, left ventricular remodeling, catheter ablation, prognosis

## Abstract

**Background:**

Left ventricular (LV) geometric remodeling is a key pathophysiological feature in heart failure with preserved ejection fraction (HFpEF), yet its prognostic implications among patients with concomitant atrial fibrillation (AF) remain unclear.

**Methods:**

In this prospective multicenter China-AF cohort study, we categorized baseline LV geometry as normal, eccentric hypertrophy, concentric remodeling, or concentric hypertrophy based on left ventricular mass index (LVMI) and relative wall thickness (RWT). The primary endpoint was a composite of cardiovascular death, thromboembolism, and major bleeding. Secondary outcomes included all-cause death and individual components. Associations were assessed using multivariable Cox regression.

**Results:**

A total of 1,691 patients were included, with a median follow-up of 4.8 years. Abnormal LV geometry was present in 50.9% of patients. Concentric remodeling (adjusted HR [aHR] 1.53, 1.17–2.01) and concentric hypertrophy (aHR 1.48, 1.10–1.99) were independently associated with higher primary endpoint risk, with concentric hypertrophy also associated with increased cardiovascular mortality and thromboembolism. Catheter ablation was associated with a lower risk of the primary outcome, with the lowest point estimate observed in the concentric remodeling subgroup (aHR 0.29, 95% CI 0.12–0.74); however, no significant interaction by LV geometry was detected (*P* for interaction = 0.147). Neither renin-angiotensin-aldosterone system inhibitors (RAASi) nor beta-blockers demonstrated benefit across geometry subtypes.

**Conclusion:**

LV geometric patterns provide meaningful prognostic stratification in patients with concomitant AF and HFpEF. Concentric remodeling and concentric hypertrophy were associated with higher risks of the primary outcome and cardiovascular mortality, whereas thromboembolic risk was most evident in concentric hypertrophy. The association between catheter ablation and a lower risk of the primary outcome in the concentric remodeling subgroup should be interpreted cautiously, given the observational design and the absence of a significant interaction by LV geometry. Further studies are warranted to validate potential phenotype-guided treatment strategies in this population.

**Clinical trial registration:**

URL: clinicaltrials.gov/study/NCT06987825, Identifier NCT06987825.

## 1 Introduction

The growing global burden of atrial fibrillation (AF) presents a pressing challenge to modern cardiovascular care, driven by demographic aging and the proliferation of cardiometabolic risk factors (1). AF-related morbidity, healthcare expenditure, and mortality are predominantly attributable to its complications, particularly stroke and heart failure (HF) (2). Contemporary epidemiological evidence from a nationwide Danish cohort reveals HF as the most prevalent complication of AF, with lifetime incidence approximately two in five in affected individuals (3). Notably, heart failure with preserved ejection fraction (HFpEF) has emerged as the dominant HF phenotype among AF patients, a clinical convergence driven by shared pathophysiological pathways and overlapping risk profiles (4). Metabolic derangements are central to this interplay, as they concurrently drive atrial myopathy and ventricular diastolic dysfunction through mechanisms that involve chronic inflammation, endothelial dysregulation, and myocardial fibrosis (5). Given the accelerating prevalence of AF-HFpEF coexistence, identifying phenotypic predictors of adverse outcomes in this population constitutes an urgent clinical priority (6).

Cardiac structural remodeling is a central mechanism underlying AF-HFpEF progression, in which left ventricular (LV) geometric patterns serve as quantifiable indices of adaptive and maladaptive myocardial responses (7). Current classification systems categorize LV geometry through assessment of mass and relative wall thickness (RWT), delineating four distinct phenotypes: normal geometry, concentric remodeling, eccentric hypertrophy, and concentric hypertrophy. Concentric hypertrophy is consistently associated with a poor prognosis in general HFpEF cohorts (8–10). However, its prognostic relevance and treatment implications remain unestablished in the AF-HFpEF population. This knowledge gap is particularly concerning given that AF imposes unique hemodynamic changes, including irregular ventricular filling patterns, rate-related myocardial injury, and neurohormonal activation, that may fundamentally influence the remodeling process (11). Despite increasing recognition of AF-HFpEF as a distinct clinical entity, systematic evaluation of LV geometric patterns in this population remains conspicuously absent from contemporary literature.

To address this critical evidence gap, we conducted a multicenter prospective analysis leveraging the Chinese Atrial Fibrillation (China-AF) registry. Using standardized echocardiographic criteria to classify baseline LV geometry, this study aimed to advance phenotypic stratification and inform targeted therapeutic strategies for this clinical entity with synergistic pathophysiology.

## 2 Methods

### 2.1 Study design and population

**Figure 1:**
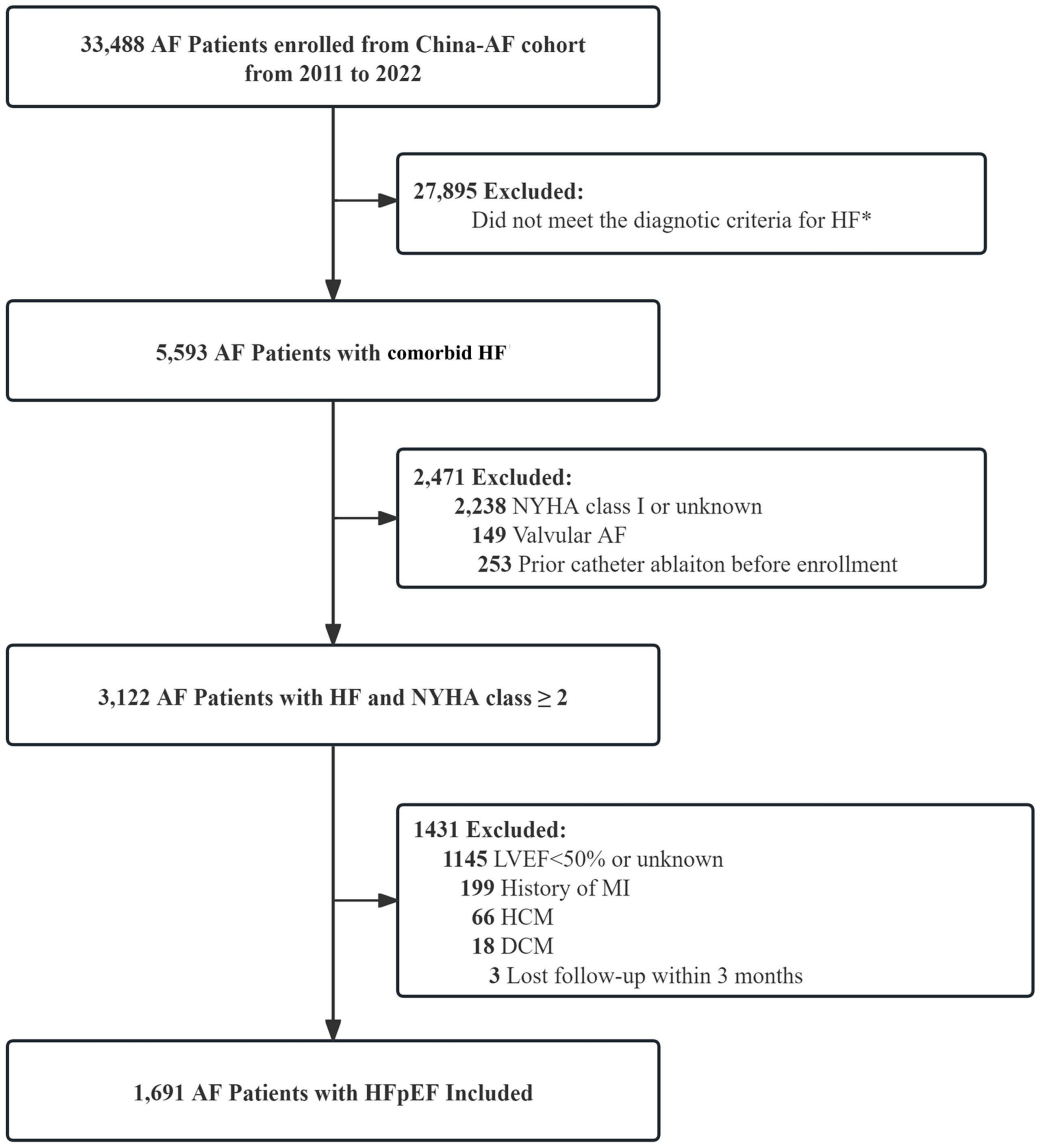
The study flow diagram. *The diagnostic criteria for HF in China-AF cohort adhered to the 2021 European Society of Cardiology (ESC) guideline for the management of heart failure. The number of patients assessed for eligibility and the reasons for exclusion. AF = atrial fibrillation; HF = heart failure; NYHA = New York Heart Association; LVEF = left ventricular ejection fraction; MI = myocardial infarction; HCM = hypertrophic cardiomyopathy; DCM = dilated cardiomyopathy; HFpEF = heart failure with preserved ejection fraction.

China-AF registry (NCT06987825) represents an ongoing prospective, multicenter, hospital-based observational cohort across 31 tertiary care centers in Beijing. Detailed rationale and design have been previously published (12). Eligible participants included adults aged 18 or older with ECG-confirmed AF and were consecutively enrolled since August 2011. The institutional review board of each participating center approved the study protocol. All participants provided written informed consent. The present study, based on the China-AF registry, specifically included patients with AF and concomitant HFpEF. In accordance with the 2021 ESC Guidelines for the diagnosis and treatment of acute and chronic heart failure, HFpEF in our study was defined by documented symptoms and/or signs of heart failure, reflected by New York Heart Association class II or higher at enrollment, baseline echocardiographic LVEF ≥50%, elevated BNP (≥105 pg./mL) or NT-proBNP (≥365 pg./mL), and echocardiographic evidence of structural and/or functional cardiac abnormalities consistent with HFpEF (Supplementary Table 1) (13). Detailed inclusion and exclusion criteria are presented in the flow diagram (Figure 1). This study adheres to the STROBE reporting guidelines for observational studies.

### 2.2 LV geometry patterns

Echocardiographic procedures were conducted in accordance with the American Society of Echocardiography (ASE) guidelines using standardized clinical protocols. We categorized LV geometry patterns by key measured parameters including left ventricular end-diastolic diameter (LVEDD), interventricular septal thickness (IVSd), posterior wall thickness (PWTd). LV mass (LVM) was calculated using the linear formula recommended by the ASE guideline: LV mass = 0.8 × (1.04([LVEDD + PWTd + IVSd]^3^ − LVEDD^3^)) + 0.6 g. LV mass index (LVMI) was determined by dividing the LV mass by the body surface area in m^2^. A cut-off of 115 g/m^2^ for males and 95 g/m^2^ for females was used to define LV hypertrophy. Relative wall thickness (RWT) was derived as: 2 × PWTd/LVEDD and increased RWT was defined as >0.42. Participants were stratified into four LV geometric patterns: normal geometry (normal LVMI and RWT ≤ 0.42), eccentric hypertrophy (LV hypertrophy and RWT ≤ 0.42), concentric remodeling (normal LVMI and RWT > 0.42), and concentric hypertrophy (LV hypertrophy and RWT > 0.42).

### 2.3 Study outcomes

The primary outcome of this study was a composite of cardiovascular death, thromboembolism event, and major bleeding. Secondary outcomes comprised all-cause death, cardiovascular death, thromboembolism, and major bleeding. Cardiovascular death included death due to myocardial infarction, heart failure, sudden cardiac death, intracranial hemorrhage, ischemic stroke, and others. The thromboembolism event included ischemic stroke and peripheral embolism other than the cerebrovascular system. Major bleeding was defined according to the International Society of Thrombosis and Haemostasis (ISTH) criteria (14). The adjudication of endpoints was conducted by an independent endpoint committee.

### 2.4 Statistical analysis

Normally distributed continuous variables were described as mean (standard deviation, SD) and compared using one-way ANOVA, whereas non-normally distributed continuous variables were presented as median (interquartile range, IQR) and compared using the Kruskal-Wallis test. Categorical variables were expressed as percentages and compared by Chi-Square test. The Kaplan–Meier curve and incidence rate per 100 person-years of each LV geometry group were generated, using log-rank tests for group comparison. The associations between LV geometry category and outcomes were evaluated by Cox proportional hazards regression models. Covariates included in the primary multivariable model were selected according to a prespecified directed acyclic graph (DAG) to define the minimal sufficient adjustment set. In this framework, baseline oral anticoagulant and antiplatelet therapy were not specified as primary confounders of the association between LV geometry and outcomes (Supplementary Figure 1). The minimal sufficient adjustment sets in the Cox models were determined using a directed acyclic graph (DAG) method (Supplementary Figure 1), including age, sex, body mass index (BMI), catheter ablation, current alcohol drinking, persistent AF, AF duration, hypertension, diabetes, coronary artery disease (CAD), chronic kidney disease (CKD), anemia, left atrial diameter (LAD), moderate to severe mitral regurgitation (MR), angiotensin-converting enzyme inhibitors/angiotensin receptor blockers (ACEI/ARB), ventricular rate control drugs (VRCD). We further calculated the adjusted hazard ratios (aHRs) for treatment strategies and primary composite outcome using Cox models in different LV geometry categories. The adjusted variables in Cox models for different treatment and primary outcome were also selected using DAGs. For renin-angiotensin-aldosterone system inhibitors (RAASi) and beta-blockers, Cox models adjusted for age, sex, systolic blood pressure (SBP), New York Heart Association (NYHA) class, CAD, LAD, eGFR, and persistent AF. For catheter ablation, Cox models adjusted for age, sex, SBP, NYHA class, LAD, estimated glomerular filtration rate (eGFR), and persistent AF. The interaction effect between treatment and LV geometry groups was assessed by adding an interaction term to the Cox models.

Sensitivity analyses were conducted to assess the robustness of the main results. We first performed an additional sensitivity analysis with further adjustment for baseline oral anticoagulant and antiplatelet therapy. Then we calculated the E-value based on the corresponding hazard ratio to assess the potential impact of unmeasured confounding on our primary results. The E-value represents the minimum strength of association that an unmeasured confounder would need to significantly influence the observed relationship between exposure and outcome (15). In addition, the Fine-Gray model was used to evaluate the associations between LV geometry and thromboembolism and major bleeding, with other deaths treated as competing risk. A two-sided *p* < 0.05 was considered statistically significant. All analyses were performed using R software (version 4.2.0; the R Foundation for Statistical Computing).

## 3 Results

### 3.1 Baseline characteristics

Among 33,488 patients with AF enrolled in China-AF cohort, a total of 1,691 AF patients with HFpEF as defined by 2021 ESC guidelines were included in final analysis (Figure 1). Participants were classified into four LV geometric patterns according to LVMI and RWT: normal geometry (*N* = 830), eccentric hypertrophy (*N* = 349), concentric remodeling (*N* = 296), and concentric hypertrophy (*N* = 216). Baseline characteristics stratified by LV geometry are presented in Table 1. Briefly, patients with normal LV geometry were more likely to be male, with lower blood pressure and lower NYHA class. The concentric hypertrophy group demonstrated the highest prevalence of diabetes mellitus and CKD, followed by eccentric hypertrophy, concentric remodeling, and normal geometry groups. A higher proportion of moderate to severe MR was noted in patients with eccentric hypertrophy. Patients with normal geometry or concentric remodeling were more likely to receive catheter ablation. No significant differences were observed in the use of oral anticoagulants or antiplatelet agents between groups. By contrast, ACEI/ARB, VRCD, and digoxin were more commonly prescribed in both concentric and eccentric hypertrophy groups.

**Table 1:** Baseline characteristics.

Variables	Normal geometry (*N* = 830)	Eccentric hypertrophy (*N* = 349)	Concentric remodeling (*N* = 296)	Concentric hypertrophy (*N* = 216)	*p* value
Age, years	72.3 ± 10.8	71.6 ± 9.8	72.0 ± 10.5	72.6 ± 10.3	0.042
Female	366 (44.1)	222 (63.6)	136 (45.9)	150 (69.4)	< 0.001
BMI, kg/m^2^	25.5 ± 3.8	25.4 ± 4.1	26.0 ± 4.1	25.5 ± 4.2	0.332
Current alcohol drinking	127 (15.3)	39 (11.2)	45 (15.2)	20 (9.3)	0.049
Current smoking	113 (13.6)	37 (10.6)	42 (14.2)	23 (10.6)	0.331
Persistent AF	479 (57.7)	203 (58.2)	180 (60.8)	111 (51.4)	0.195
AF duration, years	3.7 (0.9, 8.4)	3.8 (1.0, 8.0)	3.6 (1.1, 6.9)	3.0 (0.8, 9.6)	0.860
Catheter ablation	202 (24.3)	48 (13.8)	73 (24.7)	32 (14.8)	< 0.001
SBP, mmHg	127 ± 20	132 ± 21	130 ± 23	136 ± 21	< 0.001
DBP, mmHg	79 ± 15	81 ± 15	82 ± 16	81 ± 15	0.006
HR, bpm	82 ± 22	84 ± 24	86 ± 21	87 ± 23	0.006
NYHA class					< 0.001
II	626 (75.4)	211 (60.5)	192 (64.9)	128 (59.3)	
III	154 (18.6)	97 (27.8)	77 (26)	60 (27.8)	
IV	50 (6)	41 (11.7)	27 (9.1)	28 (13)	
Comorbidity
CAD	142 (17.1)	71 (20.3)	50 (16.9)	50 (23.1)	0.141
Hypertension	616 (74.2)	292 (83.7)	253 (85.5)	184 (85.2)	< 0.001
Controlled	135 (16.3)	58 (16.6)	54 (18.2)	31 (14.4)	0.703
Uncontrolled	481 (58.0)	234 (67.0)	199 (67.2)	153 (70.8)	< 0.001
Diabetes	227 (27.3)	114 (32.7)	99 (33.4)	80 (37)	0.018
CKD	42 (5.1)	32 (9.2)	18 (6.1)	21 (9.7)	0.016
Anemia	124 (14.9)	69 (19.8)	49 (16.6)	40 (18.5)	0.192
PAD	10 (1.2)	2 (0.6)	9 (3)	3 (1.4)	0.055
Hyperlipidemia	435 (52.4)	183 (52.4)	153 (51.7)	122 (56.5)	0.708
CHA₂DS₂-VA score	3 (2, 4)	3 (2, 4)	4 (3, 5)	4 (2, 5)	< 0.001
HAS-BLED score	2 (2, 3)	3 (2, 4)	3 (2, 3)	3 (2, 4)	< 0.001
Echocardiography
LAD, mm	43.1 ± 6.5	46.5 ± 7.5	43.0 ± 6.9	44.5 ± 7.2	< 0.001
IVS, mm	9.1 ± 1.2	10.4 ± 1.3	10.2 ± 1.4	11.8 ± 2.0	< 0.001
LVPW, mm	8.6 ± 1.0	9.7 ± 1.0	10.2 ± 1.0	11.6 ± 1.5	< 0.001
LVEDD, mm	48.3 ± 4.5	54.4 ± 5.3	43.0 ± 4.4	48.3 ± 4.6	< 0.001
LVM, g	147.2 ± 33.8	214.1 ± 48.4	147.0 ± 37.4	218.9 ± 64.3	< 0.001
LVMI, g/m^2^	82.4 ± 15.3	123.8 ± 23.2	82.3 ± 17.5	126.8 ± 32.8	< 0.001
RWT	0.36 ± 0.04	0.36 ± 0.04	0.48 ± 0.06	0.48 ± 0.07	< 0.001
LVEF, %	63 ± 7	60 ± 7	64 ± 6	63 ± 7	< 0.001
Moderate to severe MR	62 (7.5)	51 (14.6)	15 (5.1)	14 (6.5)	< 0.001
Medications
OAC	368 (44.3)	139 (39.8)	128 (43.2)	78 (36.1)	0.123
NOAC	110 (13.3)	35 (10)	33 (11.1)	28 (13)	0.420
Warfarin	258 (31.1)	106 (30.4)	95 (32.1)	50 (23.1)	0.116
Antiplatelet drug	360 (43.4)	161 (46.1)	124 (41.9)	107 (49.5)	0.280
ACEI/ARB	353 (42.5)	194 (55.6)	139 (47)	114 (52.8)	< 0.001
AAD	150 (18.1)	47 (13.5)	46 (15.5)	18 (8.3)	0.003
VRCD	470 (56.6)	235 (67.3)	182 (61.5)	145 (67.1)	0.001
Beta-blockers	408 (49.2)	191 (54.7)	150 (50.7)	123 (56.9)	0.115
CCB	50 (6)	16 (4.6)	28 (9.5)	13 (6)	0.077
Digoxin	113 (13.6)	76 (21.8)	46 (15.5)	37 (17.1)	0.006
Statin	375 (45.2)	181 (51.9)	141 (47.6)	112 (51.9)	0.111

BMI = body mass index; AF = atrial fibrillation; SBP = systolic blood pressure; DBP = diastolic blood pressure; HR = heart rate; NYHA = New York Heart Association; CAD = coronary artery disease; CKD = chronic kidney disease; PAD = peripheral artery disease; LAD = left atrium diameter; IVS = interventricular septum; LVPW = left ventricular posterior wall; LVEDD = left ventricular end-diastolic dimension; LVM = left ventricular mass; LVMI = left ventricular mass index; RWT = relative wall thickness; LVEF = left ventricular ejection fraction; MR = mitral regurgitation; OAC = oral anticoagulant; NOAC = novel oral anticoagulant; AAD = anti-arrhythmic drug, including sotalol, propafenone, or amiodarone; VRCD = ventricular rate control drug, including beta-blockers, digoxin or non-dihydropyridine calcium channel blockers; ACEI = angiotensin-converting enzyme inhibitors; ARB = angiotensin receptor blockers; CCB = calcium channel blocker.

### 3.2 Association between LV geometry and outcomes

During a median follow-up of 4.8 years (IQR: 2.0–7.5 years), a total of 414 primary composite outcomes, 493 all-cause deaths, 261 cardiovascular deaths, 183 thromboembolism events, and 59 major bleeding occurred. When analyzed as continuous variables, each SD increase in LVMI and RWT was independently associated with increased risk of composite outcome, all-cause death, and cardiovascular death (Table 2). Each SD increase in RWT was also significantly associated with increased incidence of thromboembolism events. When categorized as a binary variable, the adjusted association between LVMI hypertrophy and composite outcome was not significant (aHR: 1.21, 95% CI: 0.98–1.50, *p* = 0.074), while significant correlations with all-cause death (aHR: 1.41, 95% CI: 1.16–1.72, *p* = 0.001) and cardiovascular death (aHR: 1.39, 95% CI: 1.06–1.82, *p* = 0.015) persisted (Table 2; Supplementary Figure 2). By contrast, a larger RWT (RWT > 0.42) was consistently associated with increased incidence of composite outcome (aHR: 1.37, 95% CI: 1.12–1.69, *p* = 0.002), all-cause death (aHR: 1.35, 95% CI: 1.12–1.64, *p* = 0.002), and cardiovascular death (aHR: 1.46, 95% CI: 1.13–1.89, *p* = 0.004) (Table 2; Supplementary Figure 3). Furthermore, a significant correlation between RWT and thromboembolism event was also validated when analyzing RWT as a categorical variable (aHR: 1.40, 95% CI: 1.03–1.90, *p* = 0.031) (Table 2; Supplementary Figure 3).

**Table 2:** Associations between echo-parameters and outcomes.

Parameter	Unadjusted HR	P value	Adjusted HR^*^	*p* value
LVMI (per 1-SD increase)				
Composite outcome	1.14 (1.04–1.25)	0.004	1.12 (1.01–1.23)	0.026
All-cause death	1.24 (1.15–1.34)	<0.001	1.21 (1.12–1.32)	<0.001
Cardiovascular death	1.22 (1.10–1.36)	<0.001	1.20 (1.06–1.34)	0.003
Thromboembolism	1.07 (0.92–1.23)	0.375	1.07 (0.92–1.25)	0.376
Major bleeding	0.81 (0.60–1.09)	0.170	0.82 (0.60–1.11)	0.199
RWT (per 1-SD increase)				
Composite outcome	1.15 (1.05–1.27)	0.003	1.13 (1.03–1.24)	0.009
All-cause death	1.18 (1.09–1.28)	<0.001	1.17 (1.08–1.28)	<0.001
Cardiovascular death	1.20 (1.07–1.34)	0.002	1.19 (1.06–1.33)	0.003
Thromboembolism	1.21 (1.06–1.39)	0.005	1.19 (1.03–1.36)	0.016
Major bleeding	0.80 (0.60–1.06)	0.120	0.78 (0.58–1.05)	0.106
LVMI hypertrophy vs. No hypertrophy				
Composite outcome	1.38 (1.13–1.68)	0.001	1.21 (0.98–1.50)	0.074
All-cause death	1.49 (1.24–1.78)	<0.001	1.41 (1.16–1.72)	0.001
Cardiovascular death	1.59 (1.25–2.03)	<0.001	1.39 (1.06–1.82)	0.015
Thromboembolism	1.28 (0.95–1.72)	0.104	1.15 (0.84–1.57)	0.398
Major bleeding	0.81 (0.46–1.43)	0.471	0.80 (0.44–1.45)	0.462
RWT>0.42 vs. RWT≤0.42				
Composite outcome	1.40 (1.14–1.71)	0.001	1.37 (1.12–1.69)	0.002
All-cause death	1.32 (1.10–1.59)	0.003	1.35 (1.12–1.64)	0.002
Cardiovascular death	1.43 (1.11–1.84)	0.006	1.46 (1.13–1.89)	0.004
Thromboembolism	1.47 (1.09–1.98)	0.013	1.40 (1.03–1.90)	0.031
Major bleeding	0.86 (0.48–1.54)	0.608	0.83 (0.46–1.51)	0.546

^*^Adjusted for age, sex, BMI, catheter ablation, current alcohol drinking, persistent AF, AF duration, hypertension, diabetes, CAD, CKD, anemia, LAD, moderate to severe MR, VRCD, ACEI/ARB. LVMI = left ventricular mass index; HR = hazard ratio; RWT = relative wall thickness; BMI = body mass index; AF = atrial fibrillation; CAD = coronary artery disease; CKD = chronic kidney disease; LAD = left atrium diameter; MR = mitral regurgitation; VRCD = ventricular rate control drug; ACEI = angiotensin-converting enzyme inhibitors; ARB = angiotensin receptor blockers.

**Figure 2:**
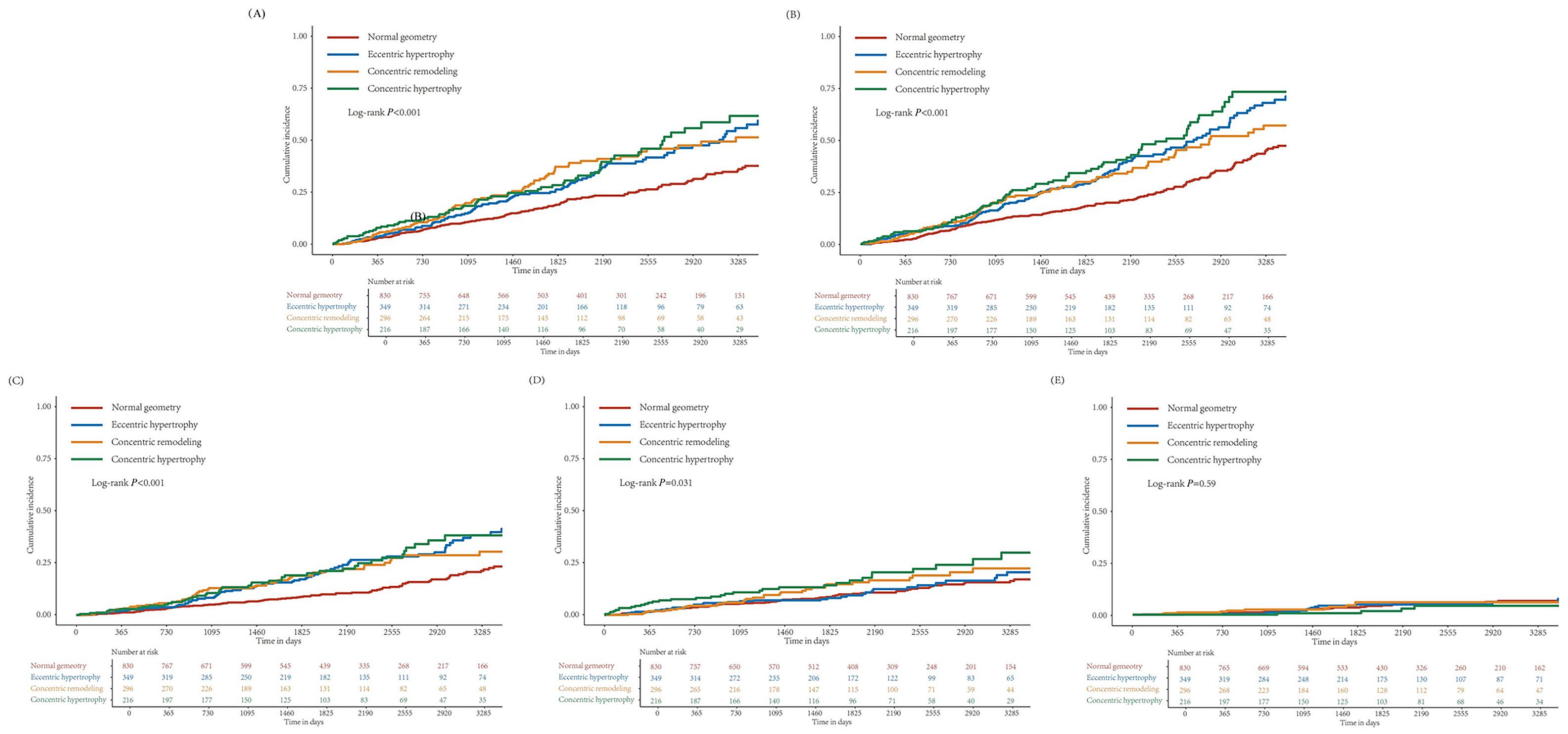
Kaplan–Meier curves for outcomes stratified by left ventricular geometries. **(A)** Composite outcome (cardiovascular death, thromboembolism, and major bleeding), **(B)** all-cause death, **(C)** cardiovascular death, **(D)** thromboembolism, and **(E)** major bleeding.

**Figure 3:**
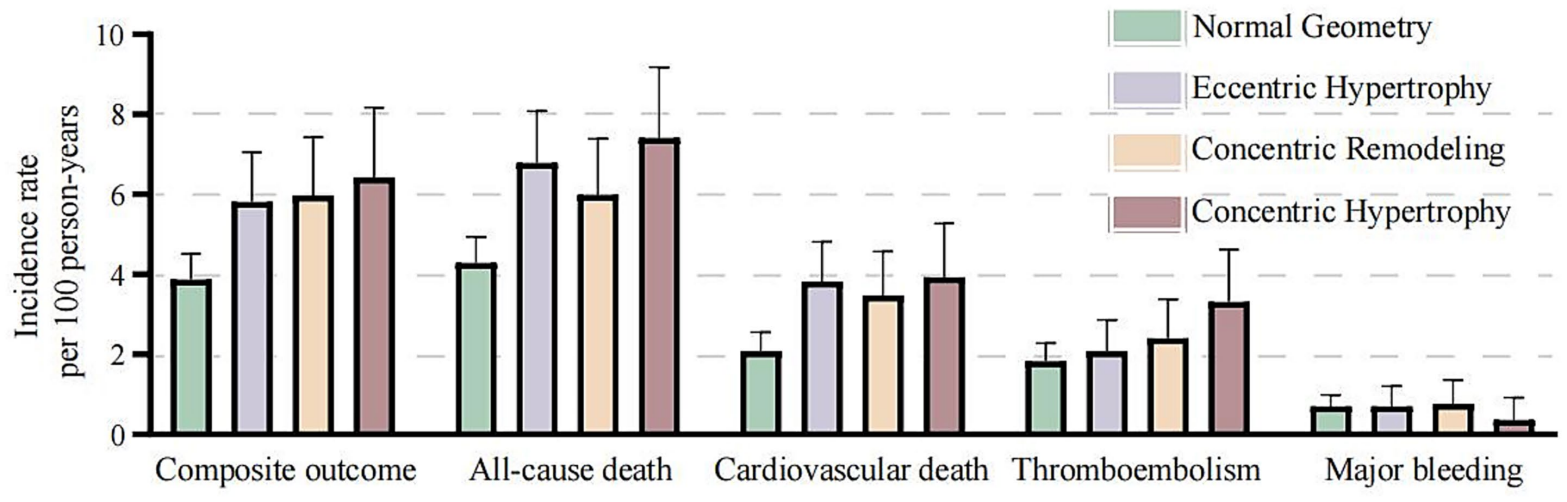
Incidence of outcomes stratified by left ventricular geometries.

The incidence and Kaplan–Meier curves of outcomes stratified by different types of LV geometry are presented in Figures 2, 3. Incidences of primary outcome, all-cause mortality, and cardiovascular mortality were all higher in patients with abnormal geometry than in those with normal geometry (Log-rank *p* < 0.001). Regarding thromboembolism events, patients with concentric hypertrophy had higher risk than those with normal geometry (Log-rank *p* = 0.031). There were no significant differences in major bleeding incidence between different LV geometry groups (Log-rank *p* = 0.590).

Table 3 presents the results of the Cox proportional hazards models. In univariate and multivariate analyses, all abnormal LV geometric categories were significantly associated with an increased risk of primary outcome, all-cause death, and cardiovascular death. Concentric remodeling demonstrated the most prominent association with primary outcome (aHR: 1.53, 95% CI: 1.17–2.01, *p* = 0.002). For all-cause death, concentric hypertrophy revealed the strongest correlation (aHR: 1.70, 95% CI: 1.30–2.22, *p* < 0.001) among abnormal geometry patterns. Concentric remodeling (aHR: 1.75, 95% CI: 1.24–2.47, *p* = 0.002) and concentric hypertrophy (aHR: 1.74, 95% CI: 1.20–2.53, *p* = 0.004) were both remarkably associated with cardiovascular death. Among three abnormal LV geometry patterns, only concentric hypertrophy was significantly associated with elevated risk of thromboembolism events (aHR: 1.58, 95% CI: 1.04–2.40, *p* = 0.031). No significant associations were observed between abnormal LV geometry and major bleeding. The competing risk analyses for embolism and major bleeding showed similar trends (Supplementary Table 2). After further adjustment for baseline oral anticoagulant and antiplatelet therapy, the overall pattern of associations between LV geometry and clinical outcomes remained largely unchanged (Supplementary Tables 3, 4). We also calculated the E-values for LV geometry and clinical outcomes in primary analysis (Supplementary Table 5). E values represent the minimum strength of association that an unmeasured confounder would need to have with both LV geometry and the outcomes to explain away the main results. The HRs of potential confounders on outcomes are listed alongside in Supplementary Table 6 for comparison with the strength of the E-values.

**Table 3:** Associations between left ventricular geometry category and outcomes.

Outcome	Unadjusted HR	*p* value	Adjusted HR^*^	*p* value
Composite outcome				
Normal geometry	Ref		Ref	
Eccentric hypertrophy	1.50 (1.17–1.92)	0.001	1.30 (1.00–1.69)	0.048
Concentric remodeling	1.56 (1.19–2.03)	0.001	1.53 (1.17–2.01)	0.002
Concentric hypertrophy	1.66 (1.25–2.21)	<0.001	1.48 (1.10–1.99)	0.009
All-cause death				
Normal geometry	Ref		Ref	
Eccentric hypertrophy	1.58 (1.26–1.97)	<0.001	1.49 (1.17–1.89)	0.001
Concentric remodeling	1.41 (1.09–1.81)	0.008	1.45 (1.13–1.88)	0.004
Concentric hypertrophy	1.73 (1.34–2.24)	<0.001	1.70 (1.30–2.22)	<0.001
Cardiovascular death				
Normal geometry	Ref		Ref	
Eccentric hypertrophy	1.84 (1.35–2.50)	<0.001	1.58 (1.14–2.19)	0.007
Concentric remodeling	1.69 (1.20–2.37)	0.003	1.75 (1.24–2.47)	0.002
Concentric hypertrophy	1.90 (1.33–2.72)	<0.001	1.74 (1.20–2.53)	0.004
Thromboembolism				
Normal geometry	Ref		Ref	
Eccentric hypertrophy	1.13 (0.76–1.67)	0.542	1.00 (0.66–1.50)	0.991
Concentric remodeling	1.31 (0.87–1.97)	0.192	1.25 (0.83–1.88)	0.293
Concentric hypertrophy	1.80 (1.21–2.70)	0.004	1.58 (1.04–2.40)	0.031
Major bleeding				
Normal geometry	Ref		Ref	
Eccentric hypertrophy	1.02 (0.54–1.96)	0.945	1.00 (0.51–1.97)	0.996
Concentric remodeling	1.13 (0.57–2.24)	0.736	1.08 (0.54–2.16)	0.836
Concentric hypertrophy	0.53 (0.19–1.49)	0.227	0.51 (0.18–1.48)	0.217

^*^Adjusted for age, sex, BMI, catheter ablation, current alcohol drinking, persistent AF, AF duration, hypertension, diabetes, CAD, CKD, anemia, LAD, moderate to severe MR, VRCD, ACEI/ARB. HR = hazard ratio; RWT = relative wall thickness; BMI = body mass index; AF = atrial fibrillation; CAD = coronary artery disease; CKD = chronic kidney disease; LAD = left atrium diameter; MR = mitral regurgitation; VRCD = ventricular rate control drug; ACEI = angiotensin-converting enzyme inhibitors; ARB = angiotensin receptor blockers.

### 3.3 Differential impact of treatment by LV geometry

**Figure 4:**
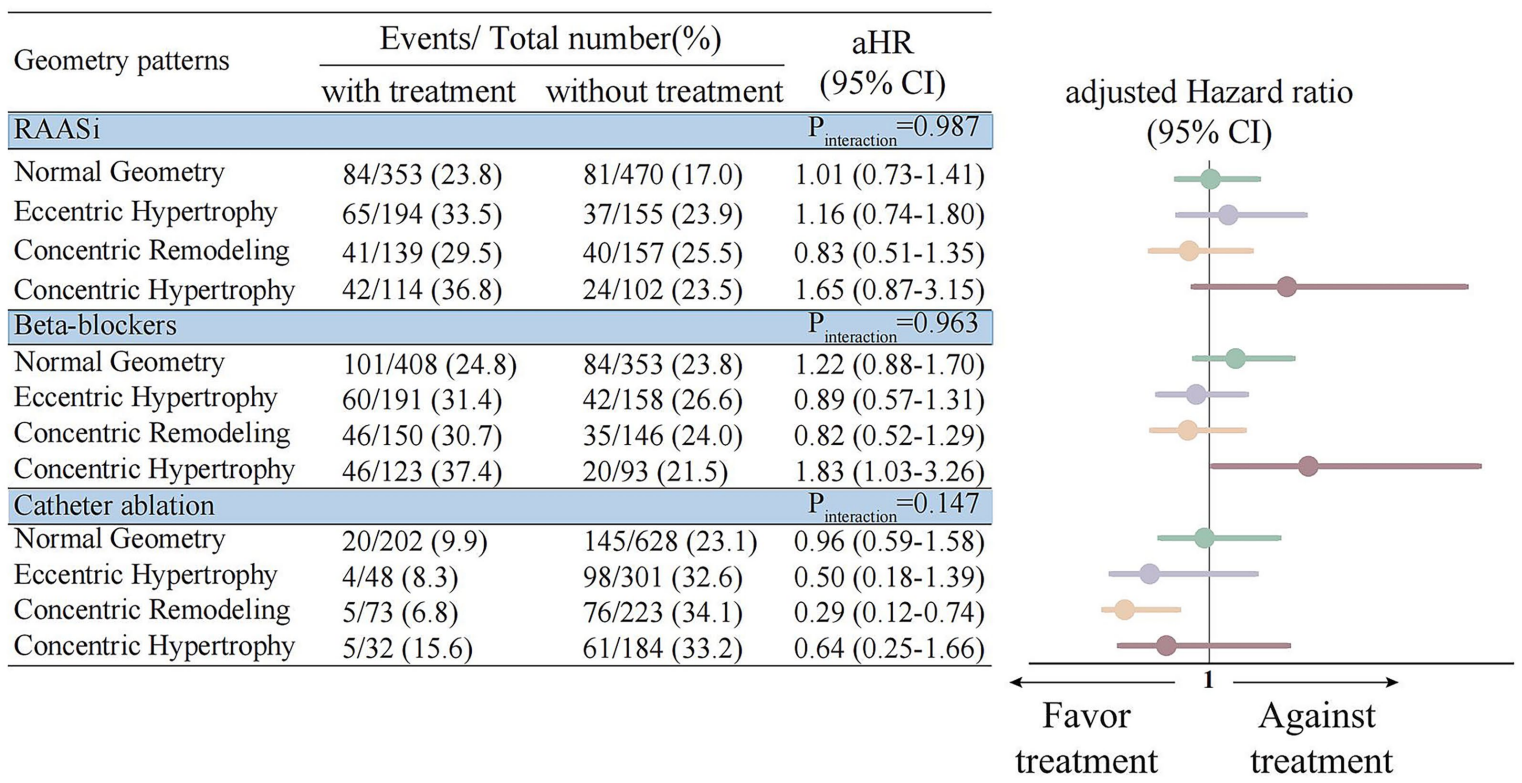
Adjusted associations of treatments and outcomes by left ventricular geometries. Adjusted HRs of different treatments with composite outcome (cardiovascular death, thromboembolism, and major bleeding) by left ventricular geometry. HR = hazard ratio; CI = confidence interval; RAASi = renin-angiotensin-aldosterone system inhibitors.

We further evaluated the association of treatment strategies targeted for HF (RAASi and beta-blockers) or AF (catheter ablation) with outcomes in LV geometry groups (Figure 4; Supplementary Table 7). There was no significant association between RAASi medication and primary outcome across LV geometry categories (*P* for interaction = 0.987). In the concentric hypertrophy subgroup, beta-blocker use was associated with a higher risk of the primary outcome (aHR 1.83, 95% CI 1.03–3.26), however, this subgroup association was based on a limited number of events and should be regarded as exploratory. No significant interaction effect was detected between beta-blockers and LV geometry (*P* for interaction = 0.963). In patients with concentric remodeling, catheter ablation was associated with a lower risk of the primary outcome (aHR 0.29, 95% CI 0.12–0.74). However, no significant interaction was detected between catheter ablation and LV geometry (*P* for interaction = 0.147).

## 4 Discussion

This multicenter prospective study delineates the clinical implications of LV geometric patterns in patients with coexisting AF and HFpEF. Three major findings warrant emphasis. First, abnormal LV geometry was observed in 50.9% of the cohort, with eccentric hypertrophy being the most prevalent abnormal pattern. Among these, concentric remodeling and concentric hypertrophy were associated with the highest risk. Second, both concentric remodeling and concentric hypertrophy were significantly associated with adverse outcomes compared with normal geometry, conferring 53 and 48% increased risk for the composite outcome, and 75 and 74% increased cardiovascular mortality, respectively. Third, our data suggest that the association of catheter ablation with outcomes may differ across LV geometry, although this interpretation should be viewed cautiously given the observational design and the lack of a statistically significant interaction (*P* for interaction = 0.147). Furthermore, no significant associations were found between the use of RAASi or beta-blockers and improved outcomes across different LV geometric patterns.

Data on LV geometry in patients with both AF and HFpEF remain limited. Prior studies have consistently shown that abnormal LV geometry is common in HFpEF. For example, in the Olmsted County community-based cohort, approximately 69% of HFpEF patients exhibited abnormal LV geometry, with concentric remodeling, concentric hypertrophy, and eccentric hypertrophy present in 27, 26, and 16% of patients, respectively (8). However, reported prevalence varies across studies, likely due to differences in study populations and inclusion criteria. In the TOPCAT trial, only 14% of participants had normal geometry (16), while in PARAGON-HF, this figure was 46% (17). Despite such variability, LV hypertrophy has consistently been linked to increased risks of mortality or heart failure hospitalization (17–19).

The prevalence of abnormal LV geometry may be lower in patients with AF. A retrospective study from Japan found that 70% of patients with AF had normal geometry, with eccentric hypertrophy, concentric remodeling, and concentric hypertrophy present in 14, 10, and 5%, respectively (11). Our study aligns with these findings: 49.1% of AF-HFpEF patients had normal geometry, and among those with abnormal patterns, eccentric hypertrophy was most common (20.6%), followed by concentric remodeling (17.5%) and concentric hypertrophy (12.8%). These results suggest that eccentric hypertrophy may predominate among AF patients, even in the context of HFpEF, contrasting with previous studies in HFpEF patients that identified concentric hypertrophy as the dominant phenotype (8, 16, 17).

Eccentric and concentric LV geometric patterns reflect adaptive responses to volume and pressure overload, respectively, and involve distinct pathophysiological mechanisms relevant to both AF and HFpEF. In our cohort, eccentric hypertrophy was frequently associated with moderate to severe mitral regurgitation, potentially contributing to increased preload. In addition, arrhythmia-related volume overload, particularly tachycardia-induced cardiomyopathy, can induce eccentric remodeling and lead to eccentric hypertrophy, which is often accompanied by a reversible decline in LV systolic function (20). In this context, both the elevated heart rate and the irregular rhythm associated with AF promote diastolic sarcoplasmic reticulum calcium leakage, which impairs excitation–contraction coupling and enhances oxidative stress, thereby facilitating adverse ventricular remodeling (21).

Hypertension remains the strongest predictor of concentric hypertrophy and is also a shared risk factor for both AF and HFpEF (22). In our study, the highest prevalence of uncontrolled hypertension was observed in patients with concentric hypertrophy, followed by those with concentric remodeling and eccentric hypertrophy. These findings are consistent with previous population-based data (23, 24). Metabolic and renal dysfunction, including obesity, insulin resistance, diabetes, and chronic kidney disease, have also been linked to concentric hypertrophy (25–27), highlighting the important contribution of neurohormonal and metabolic pathways to the interplay between AF, HFpEF, and cardiac remodeling. In animal models, concentric hypertrophy is associated with more severe myocardial fibrosis and apoptosis, as well as upregulation of signaling pathways such as MAPK kinase, *β*-arrestin-2, Akt, and calcium-handling proteins (28, 29). These molecular distinctions may underlie the poorer cardiovascular outcomes observed in patients with concentric hypertrophy in our study.

LV remodeling is a recognized therapeutic target in heart failure, particularly in patients with reduced ejection fraction (HFrEF), where RAASi and beta-blockers have demonstrated survival benefits due to their anti-remodeling properties (30). However, their role in HFpEF, especially in those with comorbid AF, remains uncertain (31). Both real-world data and randomized controlled trials suggest limited benefit from these agents in HFpEF, regardless of AF status (32–34). Consistent with prior evidence, our study did not observe significant associations of RAASi or beta-blockers with improved outcomes across different LV geometric patterns. Given the heterogeneous evidence base for these therapies in HFpEF and the absence of longitudinal information on treatment intensity and persistence in our cohort, these findings should be interpreted as neutral associations within this registry rather than definitive evidence of no therapeutic effect. The subgroup association observed for beta-blockers in patients with concentric hypertrophy should be interpreted with considerable caution. In view of the limited number of events and the non-random allocation of therapy, this estimate is particularly susceptible to residual confounding by indication and should not be interpreted as evidence of a causal harmful effect of beta-blocker therapy.

Catheter ablation is the most effective strategy for rhythm control in AF, and its benefits in HFrEF are increasingly supported by robust evidence (35–37). However, data regarding its efficacy in HFpEF are less definitive, with randomized trials and observational studies yielding mixed results (38–41). This inconsistency raises the question of which HFpEF subpopulations are most likely to benefit from ablation. Although no statistically significant interaction by LV geometry was detected, the lowest point estimate for the association between catheter ablation and the primary outcome was observed in patients with concentric remodeling, whereas the estimates in the other subgroups were more neutral. One possible explanation is that concentric remodeling represents a potentially reversible stage of cardiomyopathy, in which rhythm control may more effectively reverse atrioventricular dysfunction and support structural recovery (42). In contrast, LV hypertrophy may reflect more advanced and maladaptive remodeling, characterized by increased extracellular matrix expansion and decreased cardiomyocyte volume, indicating less reversibility (43). These structural features may help explain the less favorable estimates observed in hypertrophic phenotypes.

Overall, although the association of catheter ablation with the primary outcome appeared favorable in patients with concentric remodeling, the absence of a significant interaction precludes a definitive subgroup-specific interpretation. Accordingly, the use of LV geometry alone to guide selection for catheter ablation in AF-HFpEF was not supported by the present data. Further studies are needed to determine whether LV geometry provides clinically useful information when catheter ablation is being considered.

Several limitations should be acknowledged. First, LV geometry was defined using standard echocardiographic parameters without strain imaging, which may have limited the detection of more subtle abnormalities in myocardial function. Second, although multivariable adjustments were performed, residual confounding from unmeasured variables cannot be entirely excluded. However, an E-value of at least 1.95 would be required to fully explain away the observed associations, which suggests that a confounder of such strength would be difficult to achieve. Third, heart failure hospitalization is a clinically relevant endpoint in HFpEF, but it was not specifically captured in the China-AF registry. Therefore, although the present study demonstrates associations between LV geometry and the selected adverse outcomes, the prognostic significance of LV geometry with respect to HF-specific morbidity may not have been fully characterized. Fourth, post-baseline exposure to RAASi and beta-blockers could not be fully characterized, precluding assessment of time-varying treatment intensity and persistence. Fifth, the associations between catheter ablation and outcomes were based on an observational design, and further studies are needed to determine whether LV geometry has a role in treatment stratification. The modest *p*-value for interaction (0.147) should be interpreted with caution, as a larger sample size may be necessary to detect true effect modification. Finally, our single-country registry limits generalizability to non-Asian populations with differing AF/HFpEF etiologies.

## 5 Conclusion

In this AF-HFpEF cohort, LV geometric patterns provided important prognostic stratification. Abnormal LV geometry was associated with higher risks of the composite outcome, all-cause mortality, and cardiovascular mortality, whereas thromboembolic risk was most evident in concentric hypertrophy. Although the association of catheter ablation appeared favorable in patients with concentric remodeling, the present findings do not support a definitive subgroup-specific interpretation. Future studies are warranted to validate these findings in diverse, multinational cohorts using advanced imaging and in randomized controlled trials. Incorporating LV morphological assessment into routine evaluation may facilitate precision management for this growing and complex patient population.

## Data Availability

The data analyzed in this study is subject to the following licenses/ restrictions: the deidentified participant data are available from the corresponding author upon reasonable request.
