# Supplementary material for: Association of left ventricular geometry with outcomes and treatment response in atrial fibrillation and heart failure with preserved ejection fraction
Source: Front Med (Lausanne). 2026 May 4;13:1824509. doi: 10.3389/fmed.2026.1824509 (PMC13180614; doi:10.3389/fmed.2026.1824509)
Supplement: Supplementary file 1 [file Data_Sheet_1.docx]

**SUPPLEMENTAL MATERIAL**

**Supplemental Figure 1. Directed acyclic graph for LV geometry and outcome**

**
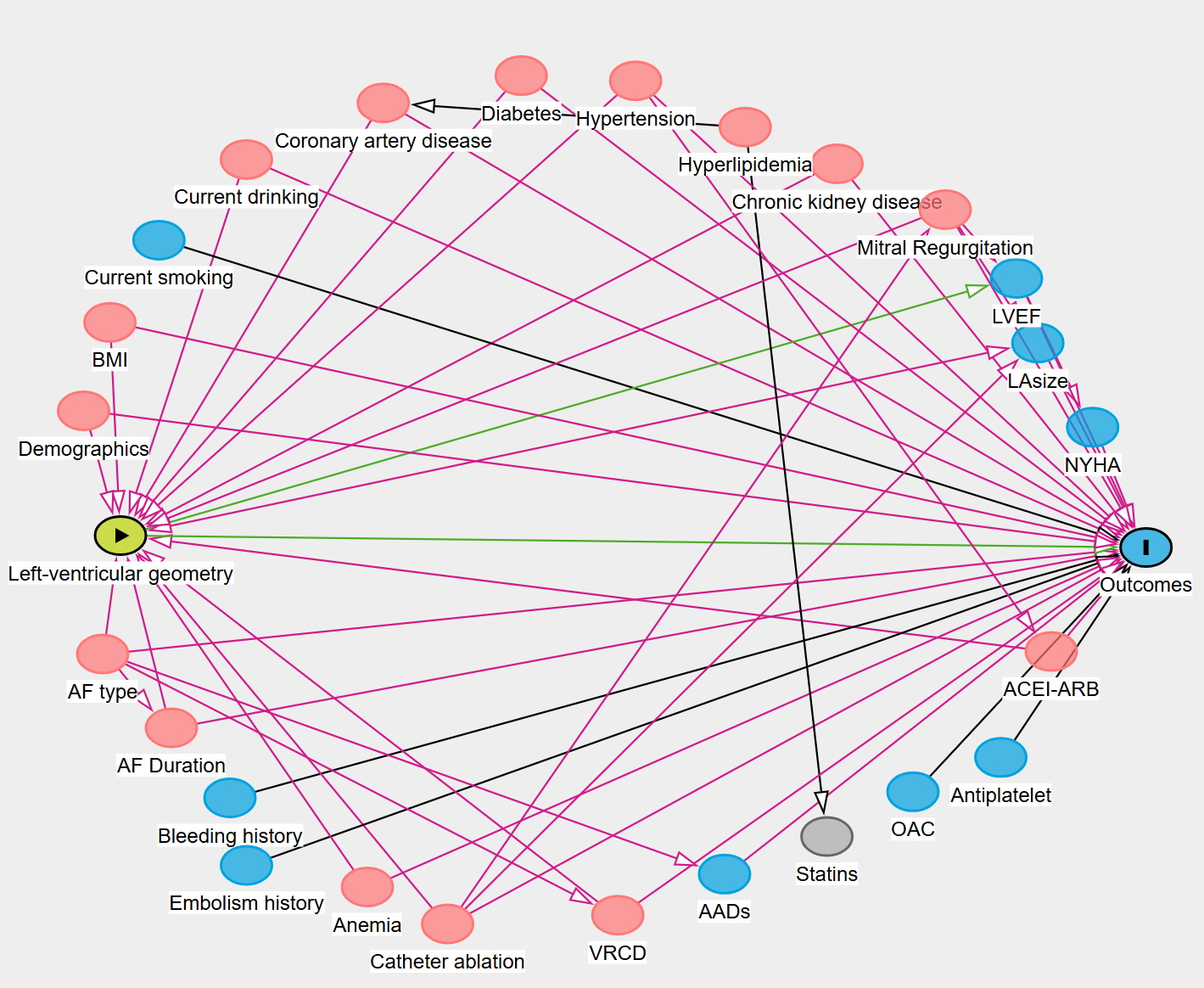
**

BMI = body mass index; AF = atrial fibrillation; LA = left atrium; LVEF = left ventricular ejection fraction; NYHA = New York Heart Association; OAC = oral anticoagulant; AAD = anti-arrhythmic drugs; VRCD = ventricular rate control drug; ACEI = angiotensin-converting enzyme inhibitors; ARB = angiotensin receptor blockers.

**Supplemental Figure 2. Incidence of Outcomes Stratified by LVMI**


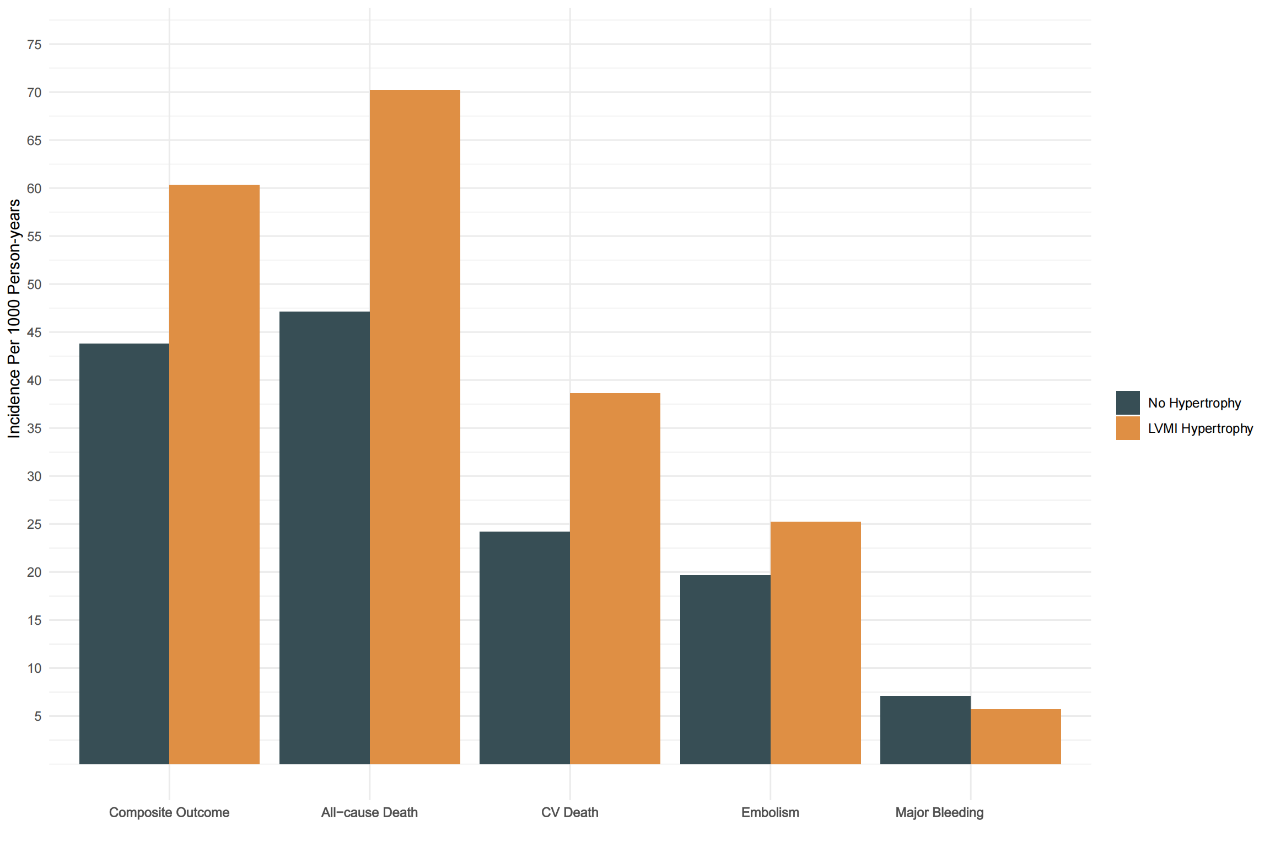


LVMI = left ventricular mass index; CV death = cardiovascular death.

**Supplemental Figure 3. Incidence of Outcomes Stratified by RWT**


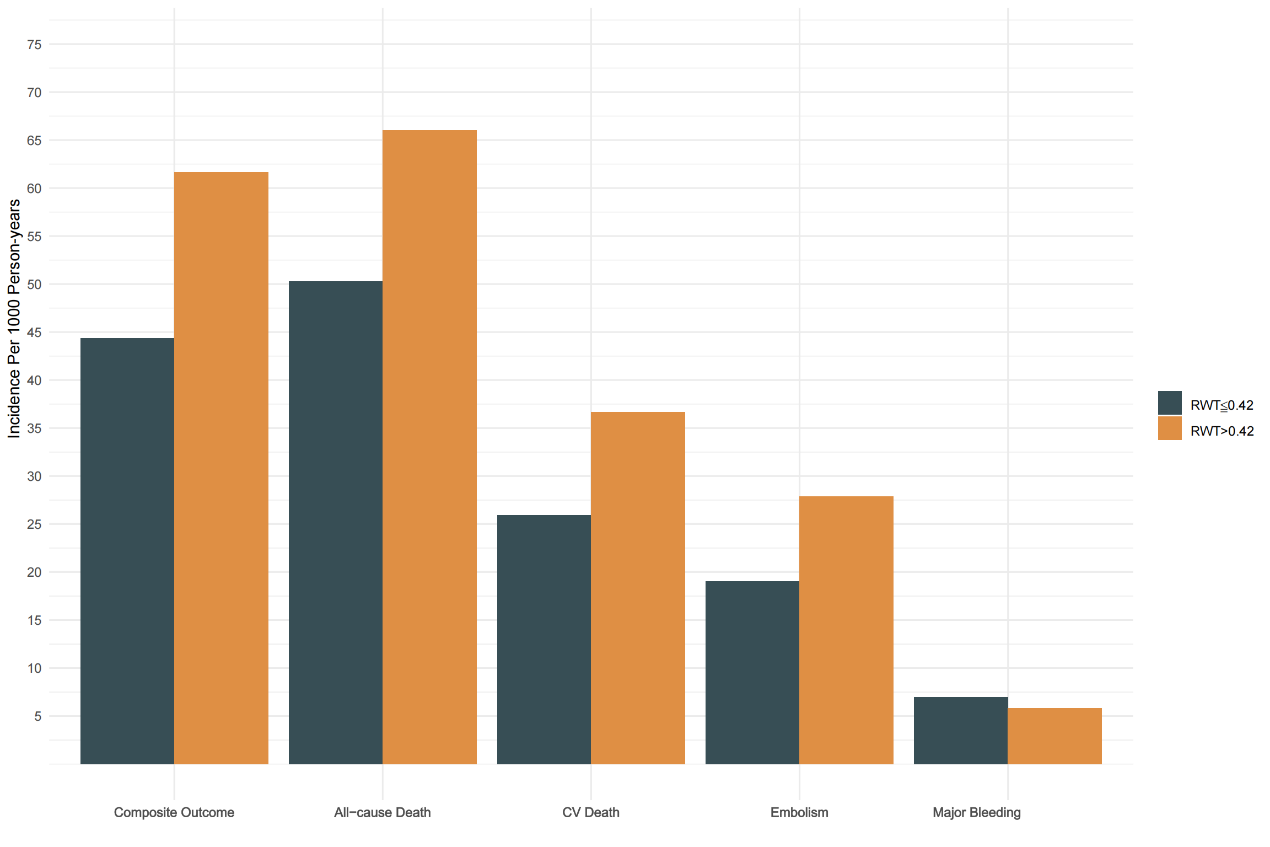


RWT = relative wall thickness; CV death = cardiovascular death.

**Supplemental Table 1.** **Diagnostic criteria for identifying heart failure with preserved ejection fraction among patients with atrial fibrillation**

| Step 1 | Step 2 | Step 3 |
| --- | --- | --- |
| Typical symptoms and signs* | LVEF ≥ 50% | High circulatory BNP/NT-proBNP level^&^ and  (1) LV/LA enlargement; or (2) left ventricular diastolic dysfunction^#^ |

*: Signs associated with heart failure include: heart enlargement, engorgement of the neck veins, hepatojugular reflux, moist rales, gallop rhythm, edema of lower extremity, hepatomegaly, pleural effusion, tachycardia.

&: The cut-off values for identifying heart failure without atrial fibrillation are BNP≥35pg/mL and NT-proBNP≥125pg/mL. However, when heart failure patients have comorbid atrial fibrillation, a higher cut-off value for BNP/NT-proBNP is needed. In this study, we have set the threshold for BNP at 105pg/mL and NT-proBNP at 365pg/mL with the objective of excluding individuals who are unlikely to have heart failure.

#: LA enlargement was defined as LA diameter > 40 mm for men and >38 for women; LV enlargement was defined as left ventricular end diastolic diameter >55 mm for men and > 50 for women.

The diastolic dysfunction was assessed by experienced sonographers according to the latest guideline available at that time.

BNP = B-type natriuretic peptide; LVEF = left ventricular ejection fraction; LV = left ventricle; LA = left atrium.

**Supplemental Table 2. Associations between LV geometry category and outcomes in competitive risk regression analysis**

| **Outcome** | **Unadjusted HR** | ***P* value** | **Adjusted HR**^*^ | ***P* value** |
| --- | --- | --- | --- | --- |
| **Thromboembolism** |  |  |  |  |
| Normal Geometry | Ref |  | Ref | Ref |
| Eccentric Hypertrophy | 1.04 (0.71-1.54) | 0.830 | 0.93 (0.62-1.40) | 0.720 |
| Concentric Remodeling | 1.21 (0.81-1.82) | 0.350 | 1.15 (0.76-1.75) | 0.510 |
| Concentric Hypertrophy | 1.63 (1.09-2.44) | 0.019 | 1.42 (0.93-2.17) | 0.100 |
| **Major Bleeding** |  |  |  |  |
| Normal Geometry | Ref |  | Ref |  |
| Eccentric Hypertrophy | 0.94 (0.49-1.80) | 0.860 | 0.93 (0.48-1.82) | 0.830 |
| Concentric Remodeling | 1.03 (0.52-2.06) | 0.930 | 0.95 (0.47-1.94) | 0.890 |
| Concentric Hypertrophy | 0.46 (0.17-1.30) | 0.150 | 0.45 (0.16-1.27) | 0.130 |

^*^ Adjusted for age, sex, BMI, catheter ablation, current alcohol drinking, persistent AF, AF duration, hypertension, diabetes, CAD, CKD, anemia, LAD, moderate to severe MR, VRCD, ACEI/ARB

HR = hazard ratio; BMI = body mass index; AF = atrial fibrillation; CAD = coronary heart disease; CKD = chronic kidney disease; LAD = left atrium diameter; MR = mitral regurgitation; VRCD = ventricular rate control drug; ACEI = angiotensin-converting enzyme inhibitors; ARB = angiotensin receptor blockers.

**Supplemental Table 3. Sensitivity analysis between echo-parameters and outcomes**

| **Parameter** | **Adjusted HR^*^** | ***P* value** |
| --- | --- | --- |
| **LVMI (per 1-SD increase)** |  |  |
| Composite Outcome | 1.11 (1.01-1.22) | 0.031 |
| All-cause death | 1.20 (1.11-1.31) | <0.001 |
| Cardiovascular death | 1.19 (1.06-1.33) | 0.004 |
| Thromboembolism | 1.07 (0.92-1.24) | 0.381 |
| Major Bleeding | 0.82 (0.60-1.12) | 0.206 |
| **RWT (per 1-SD increase)** |  |  |
| Composite Outcome | 1.12 (1.02-1.24) | 0.015 |
| All-cause death | 1.16 (1.07-1.27) | <0.001 |
| Cardiovascular death | 1.18 (1.05-1.32) | 0.005 |
| Thromboembolism | 1.18 (1.02-1.35) | 0.022 |
| Major Bleeding | 0.79 (0.59-1.06) | 0.111 |
| **LVMI Hypertrophy vs. No Hypertrophy** |  |  |
| Composite Outcome | 1.21 (0.98-1.50) | 0.074 |
| All-cause death | 1.41 (1.16-1.71) | 0.001 |
| Cardiovascular death | 1.39 (1.06-1.81) | 0.016 |
| Thromboembolism | 1.15 (0.84-1.58) | 0.379 |
| Major Bleeding | 0.80 (0.44-1.46) | 0.471 |
| **RWT>0.42 vs. RWT≤0.42** |  |  |
| Composite Outcome | 1.36 (1.11-1.66) | 0.004 |
| All-cause death | 1.32 (1.10-1.60) | 0.004 |
| Cardiovascular death | 1.43 (1.11-1.85) | 0.006 |
| Thromboembolism | 1.38 (1.02-1.88) | 0.037 |
| Major Bleeding | 0.84 (0.46-1.52) | 0.556 |

^*^ Adjusted for age, sex, BMI, catheter ablation, current alcohol drinking, persistent AF, AF duration, hypertension, diabetes, CAD, CKD, anemia, LAD, moderate to severe MR, VRCD, ACEI/ARB, OAC, antiplatelet drugs.

LVMI = left ventricular mass index; HR = hazard ratio; BMI = body mass index; RWT = relative wall thickness; AF = atrial fibrillation; CAD = coronary heart disease; CKD = chronic kidney disease; LAD = left atrium diameter; MR = mitral regurgitation; VRCD = ventricular rate control drug; ACEI = angiotensin-converting enzyme inhibitors; ARB = angiotensin receptor blockers; OAC = oral anticoagulant.

**Supplemental Table 4. Sensitivity analysis between left ventricular geometry category and outcomes**

| **Outcome** | **Adjusted HR**^*^ | ***P* value** |
| --- | --- | --- |
| **Composite Outcome** |  |  |
| Normal Geometry | Ref |  |
| Eccentric Hypertrophy | 1.31 (1.01-1.70) | 0.044 |
| Concentric Remodeling | 1.52 (1.16-1.99) | 0.002 |
| Concentric Hypertrophy | 1.46 (1.09-1.96) | 0.012 |
| **All-cause death** |  |  |
| Normal Geometry | Ref |  |
| Eccentric Hypertrophy | 1.49 (1.18-1.90) | 0.001 |
| Concentric Remodeling | 1.43 (1.10-1.84) | 0.007 |
| Concentric Hypertrophy | 1.66 (1.27-2.18) | <0.001 |
| **Cardiovascular death** |  |  |
| Normal Geometry | Ref |  |
| Eccentric Hypertrophy | 1.58 (1.14-2.20) | 0.006 |
| Concentric Remodeling | 1.71 (1.21-2.42) | 0.002 |
| Concentric Hypertrophy | 1.70 (1.17-2.48) | 0.005 |
| **Thromboembolism** |  |  |
| Normal Geometry | Ref |  |
| Eccentric Hypertrophy | 1.02 (0.68-1.53) | 0.939 |
| Concentric Remodeling | 1.25 (0.83-1.88) | 0.288 |
| Concentric Hypertrophy | 1.56 (1.03-2.37) | 0.036 |
| **Major Bleeding** |  |  |
| Normal Geometry | Ref |  |
| Eccentric Hypertrophy | 1.00 (0.51-1.98) | 0.989 |
| Concentric Remodeling | 1.08 (0.54-2.18) | 0.822 |
| Concentric Hypertrophy | 0.52 (0.18-1.49) | 0.221 |

^*^ Adjusted for age, sex, BMI, catheter ablation, current alcohol drinking, persistent AF, AF duration, hypertension, diabetes, CAD, CKD, anemia, LAD, moderate to severe MR, VRCD, ACEI/ARB, OAC, antiplatelet drugs.

HR = hazard ratio; BMI = body mass index; RWT = relative wall thickness; AF = atrial fibrillation; CAD = coronary heart disease; CKD = chronic kidney disease; LAD = left atrium diameter; MR = mitral regurgitation; VRCD = ventricular rate control drug; ACEI = angiotensin-converting enzyme inhibitors; ARB = angiotensin receptor blockers; OAC = oral anticoagulant.

**Supplemental Table 5. Assessment of the impact of unmeasured confounders using E-values***

| **Outcome** | **E value for**  **point estimation** | **E value for**  **confidence interval** |
| --- | --- | --- |
| **Composite Outcome** |  |  |
| Normal Geometry | Ref | Ref |
| Eccentric Hypertrophy | 1.69 | 1 |
| Concentric Remodeling | 2.02 | 1.47 |
| Concentric Hypertrophy | 1.95 | 1.34 |
| **All-cause death** |  |  |
| Normal Geometry | Ref | Ref |
| Eccentric Hypertrophy | 1.96 | 1.47 |
| Concentric Remodeling | 1.91 | 1.4 |
| Concentric Hypertrophy | 2.24 | 1.69 |
| **Cardiovascular death** |  |  |
| Normal Geometry | Ref | Ref |
| Eccentric Hypertrophy | 2.09 | 1.42 |
| Concentric Remodeling | 2.31 | 1.59 |
| Concentric Hypertrophy | 2.29 | 1.53 |
| **Thromboembolism** |  |  |
| Normal Geometry | Ref | Ref |
| Eccentric Hypertrophy | 1 | 1 |
| Concentric Remodeling | 1.81 | 1 |
| Concentric Hypertrophy | 2.54 | 1.24 |
| **Major bleeding** |  |  |
| Normal Geometry | Ref | Ref |
| Eccentric Hypertrophy | 1 | 1 |
| Concentric Remodeling | 1.37 | 1 |
| Concentric Hypertrophy | 3.33 | 1 |

*E-value was calculated through the hazard ratios and 95% CI from the adjusted Cox proportional hazard model analysis.

**Supplemental Table 6. Hazard ratios for the potential confounders in the primary analysis**

| **Covariates/outcomes** | **Primary Outcome** | | **All-cause death** | | **Cardiovascular death** | | **Thromboembolism** | | **Major Bleeding** | |
| --- | --- | --- | --- | --- | --- | --- | --- | --- | --- | --- |
|  | HR (95% CI) | *P*-value | HR (95% CI) | *P*-value | HR (95% CI) | *P*-value | HR (95% CI) | *P*-value | HR (95% CI) | *P*-value |
| Age, y | 1.05 (1.04-1.06) | <0.001 | 1.11 (1.09-1.12) | <0.001 | 1.1 (1.08-1.12) | <0.001 | 1.02 (1-1.03) | 0.024 | 1.03 (1-1.05) | 0.077 |
| Female sex | 0.77 (0.63-0.94) | 0.009 | 0.97 (0.81-1.16) | 0.726 | 0.78 (0.61-0.99) | 0.045 | 0.72 (0.54-0.97) | 0.033 | 0.85 (0.51-1.43) | 0.547 |
| Body mass index, kg/m^2^ | 0.96 (0.94-0.99) | 0.004 | 0.93 (0.91-0.96) | <0.001 | 0.94 (0.91-0.97) | <0.001 | 0.99 (0.95-1.03) | 0.61 | 0.97 (0.9-1.03) | 0.313 |
| Current drinker | 0.64 (0.46-0.88) | 0.006 | 0.6 (0.45-0.81) | 0.001 | 0.5 (0.32-0.78) | 0.002 | 0.69 (0.43-1.1) | 0.118 | 0.86 (0.41-1.82) | 0.701 |
| Current smoker | 0.86 (0.63-1.16) | 0.326 | 0.94 (0.71-1.23) | 0.634 | 0.96 (0.67-1.39) | 0.843 | 0.73 (0.45-1.18) | 0.199 | 1.08 (0.51-2.27) | 0.847 |
| Systolic blood pressure, mmHg | 1.01 (1-1.01) | 0.001 | 1.01 (1-1.01) | 0.025 | 1.01 (1-1.02) | 0.004 | 1.01 (1-1.02) | 0.018 | 0.99 (0.97-1) | 0.175 |
| Diastolic blood pressure, mmHg | 1 (0.99-1.01) | 0.861 | 0.99 (0.98-1) | 0.016 | 1 (0.99-1.01) | 0.476 | 1 (0.99-1.02) | 0.497 | 1 (0.98-1.02) | 0.854 |
| Persistent AF | 1.26 (1.04-1.54) | 0.02 | 1.22 (1.02-1.45) | 0.033 | 1.33 (1.03-1.7) | 0.026 | 1.13 (0.84-1.51) | 0.416 | 1.92 (1.11-3.33) | 0.019 |
| AF duration,y | 1.01 (1-1.02) | 0.136 | 1.02 (1.01-1.04) | <0.001 | 1.02 (1.01-1.04) | 0.003 | 1 (0.98-1.02) | 0.916 | 0.97 (0.93-1.02) | 0.228 |
| Coronary artery disease | 0.99 (0.77-1.27) | 0.91 | 1.08 (0.87-1.36) | 0.476 | 1.01 (0.74-1.39) | 0.935 | 0.95 (0.65-1.39) | 0.799 | 1.16 (0.61-2.18) | 0.651 |
| Hypertension | 1.45 (1.11-1.88) | 0.006 | 1.23 (0.98-1.55) | 0.074 | 1.5 (1.07-2.1) | 0.018 | 1.45 (0.97-2.15) | 0.068 | 1.35 (0.68-2.66) | 0.393 |
| Diabetes | 1.47 (1.2-1.79) | <0.001 | 1.55 (1.3-1.86) | <0.001 | 1.75 (1.37-2.24) | <0.001 | 1.31 (0.97-1.78) | 0.082 | 1.64 (0.97-2.77) | 0.065 |
| CKD | 2.24 (1.66-3.03) | <0.001 | 2.51 (1.92-3.29) | <0.001 | 3.02 (2.14-4.27) | <0.001 | 1.53 (0.9-2.6) | 0.115 | 1.08 (0.34-3.46) | 0.895 |
| Anemia | 1.64 (1.3-2.06) | <0.001 | 2.18 (1.79-2.65) | <0.001 | 2.24 (1.72-2.93) | <0.001 | 1 (0.67-1.49) | 0.998 | 1.02 (0.5-2.08) | 0.949 |
| Hyperlipidemia | 0.87 (0.72-1.06) | 0.165 | 0.72 (0.6-0.85) | <0.001 | 0.76 (0.6-0.97) | 0.03 | 1.29 (0.96-1.73) | 0.096 | 1.22 (0.72-2.06) | 0.452 |
| LVEF, % | 0.99 (0.97-1) | 0.071 | 0.99 (0.98-1.01) | 0.327 | 0.99 (0.97-1.01) | 0.275 | 0.99 (0.96-1.01) | 0.177 | 1.03 (0.99-1.06) | 0.165 |
| Left atrial diameter, mm | 0.99 (0.97-1) | 0.071 | 1.03 (1.02-1.04) | <0.001 | 1.03 (1.02-1.05) | <0.001 | 1.01 (0.99-1.03) | 0.535 | 0.99 (0.95-1.03) | 0.543 |
| OAC | 0.58 (0.47-0.72) | <0.001 | 0.48 (0.39-0.58) | <0.001 | 0.46 (0.35-0.61) | <0.001 | 0.65 (0.48-0.89) | 0.006 | 1.12 (0.67-1.88) | 0.655 |
| Antiplatelet drugs | 1.49 (1.22-1.81) | <0.001 | 1.57 (1.31-1.88) | <0.001 | 1.59 (1.24-2.04) | <0.001 | 1.52 (1.14-2.04) | 0.005 | 0.91 (0.55-1.52) | 0.728 |
| AAD | 0.59 (0.44-0.81) | 0.001 | 0.52 (0.38-0.7) | <0.001 | 0.38 (0.24-0.61) | <0.001 | 0.85 (0.56-1.28) | 0.433 | 0.7 (0.33-1.51) | 0.366 |
| VRCD | 1.25 (1.01-1.55) | 0.043 | 1.22 (1-1.48) | 0.049 | 1.35 (1.02-1.78) | 0.035 | 1.29 (0.93-1.78) | 0.123 | 0.7 (0.41-1.19) | 0.188 |
| Beta-blockers | 1.18 (0.97-1.44) | 0.099 | 0.92 (0.77-1.10) | 0.366 | 1.15 (0.89-1.47) | 0.280 | 1.31 (0.97-1.78) | 0.076 | 0.99 (0.59-1.66) | 0.965 |
| ACEI/ARB | 1.22 (1.01-1.48) | 0.044 | 1.08 (0.91-1.29) | 0.381 | 1.24 (0.97-1.58) | 0.091 | 1.21 (0.9-1.62) | 0.202 | 1.16 (0.69-1.94) | 0.566 |
| Calcium-channel blocker | 0.84 (0.55-1.26) | 0.396 | 0.89 (0.61-1.29) | 0.532 | 0.78 (0.45-1.34) | 0.364 | 0.86 (0.47-1.59) | 0.633 | 0.46 (0.11-1.9) | 0.286 |
| Digoxin | 1.64 (1.31-2.06) | <0.001 | 1.87 (1.53-2.28) | <0.001 | 2.17 (1.66-2.83) | <0.001 | 1.28 (0.89-1.84) | 0.184 | 0.76 (0.36-1.61) | 0.476 |
| Statin | 1.18 (0.97-1.43) | 0.091 | 1.03 (0.87-1.24) | 0.704 | 1.21 (0.95-1.55) | 0.119 | 1.05 (0.78-1.4) | 0.745 | 1.04 (0.62-1.73) | 0.888 |

This table is provided for comparison with the magnitude of the E-value

HR = hazard ratio; CI = confidence interval; AF = atrial fibrillation; CKD = chronic kidney disease; LVEF = left ventricular ejection fraction; OAC = oral anticoagulant; AAD = anti-arrhythmic drugs; VRCD = ventricular rate control drug; ACEI = angiotensin-converting enzyme inhibitors; ARB = angiotensin receptor blockers.

**Supplemental Table 7. Association between treatment and clinical outcomes stratified by left ventricular geometric patterns**

|  | Events (%) | | adjusted HR  (95% CI) | P-value | P for interaction |
| --- | --- | --- | --- | --- | --- |
|  | With treatment | Without treatment |  |  |  |
| RAASi |  |  |  |  |  |
| **All-cause death** |  |  |  |  | 0.763 |
| Normal geometry | 91 (25.8) | 103 (21.6) | 1.06 (0.78-1.43) | 0.711 |  |
| Eccentric hypertrophy | 80 (41.2) | 48 (31.0) | 1.16 (0.78-1.73) | 0.466 |  |
| Concentric remodeling | 43 (30.9) | 45 (28.7) | 0.77 (0.49-1.21) | 0.252 |  |
| Concentric hypertrophy | 49 (43.0) | 34 (33.3) | 0.83 (0.50-1.39) | 0.483 |  |
| **Cardiovascular death** |  |  |  |  | 0.868 |
| Normal geometry | 46 (13.0) | 48 (10.1) | 1.00 (0.65-1.55) | 0.991 |  |
| Eccentric hypertrophy | 46 (23.7) | 26 (16.8) | 1.05 (0.61-1.79) | 0.869 |  |
| Concentric remodeling | 27 (19.4) | 24 (15.3) | 0.93 (0.50-1.70) | 0.802 |  |
| Concentric hypertrophy | 29 (25.4) | 15 (14.7) | 1.20 (0.56-2.54) | 0.639 |  |
| **Thromboembolism** |  |  |  |  | 0.737 |
| Normal geometry | 40 (11.3) | 39 (8.2) | 0.92 (0.57-1.46) | 0.712 |  |
| Eccentric hypertrophy | 23 (11.9) | 14 (9.0) | 1.26 (0.60-2.64) | 0.536 |  |
| Concentric remodeling | 18 (12.9) | 15 (9.6) | 1.02 (0.47-2.24) | 0.956 |  |
| Concentric hypertrophy | 21 (18.4) | 13 (12.7) | 1.95 (0.77-4.97) | 0.160 |  |
| **Major bleeding** |  |  |  |  |  |
| Normal geometry | 16 (4.5) | 15 (3.1) | 1.08 (0.51-2.32) | 0.837 |  |
| Eccentric hypertrophy | 8 (4.1) | 5 (3.2) | 1.25 (0.34-4.61) | 0.741 |  |
| Concentric remodeling | 6 (4.3) | 5 (3.2) | 0.77 (0.21-2.83) | 0.689 |  |
| Concentric hypertrophy | 3 (2.6) | 1 (1.0) | - |  |  |
| Beta-blockers |  |  |  |  |  |
| **All-cause death** |  |  |  |  | 0.256 |
| Normal geometry | 112 (27.5) | 82 (19.4) | 1.06 (0.78-1.43) | 0.711 |  |
| Eccentric hypertrophy | 69 (36.1) | 59 (37.3) | 0.65 (0.45-0.95) | 0.024 |  |
| Concentric remodeling | 46 (30.7) | 42 (28.8) | 0.70 (0.45-1.09) | 0.112 |  |
| Concentric hypertrophy | 49 (39.8) | 34 (36.6) | 0.92 (0.57-1.50) | 0.734 |  |
| **Cardiovascular death** |  |  |  |  | 0.873 |
| Normal geometry | 60 (14.7) | 34 (8.1) | 1.35 (0.86-2.11) | 0.190 |  |
| Eccentric hypertrophy | 40 (20.9) | 32 (20.3) | 0.68 (0.42-1.12) | 0.130 |  |
| Concentric remodeling | 29 (19.3) | 22 (15.1) | 0.83 (0.47-1.48) | 0.526 |  |
| Concentric hypertrophy | 31 (25.2) | 13 (14.0) | 1.73 (0.83-3.59) | 0.143 |  |
| **Thromboembolism** |  |  |  |  | 0.874 |
| Normal geometry | 49 (12.0) | 30 (7.1) | 1.21 (0.75-1.96) | 0.436 |  |
| Eccentric hypertrophy | 23 (12.0) | 14 (8.9) | 1.09 (0.54-2.18) | 0.812 |  |
| Concentric remodeling | 21 (14.0) | 12 (8.2) | 1.10 (0.53-2.29) | 0.798 |  |
| Concentric hypertrophy | 23 (18.7) | 11 (11.8) | 1.79 (0.80-4.04) | 0.159 |  |
| **Major bleeding** |  |  |  |  |  |
| Normal geometry | 17 (4.2) | 14 (3.3) | 0.96 (0.45-2.03) | 0.909 |  |
| Eccentric hypertrophy | 8 (4.2) | 5 (3.2) | 0.99 (0.28-3.45) | 0.985 |  |
| Concentric remodeling | 6 (4.0) | 5 (3.4) | 0.82 (0.21-3.13) | 0.769 |  |
| Concentric hypertrophy | 4 (3.3) | 0 | - |  |  |
| Catheter ablation |  |  |  |  |  |
| **All-cause death** |  |  |  |  | 0.953 |
| Normal geometry | 12 (5.9) | 182 (29.0) | 0.72 (0.39-1.34) | 0.299 |  |
| Eccentric hypertrophy | 3 (6.3) | 125 (41.5) | 0.48 (0.15-1.58) | 0.231 |  |
| Concentric remodeling | 5 (6.8) | 83 (37.2) | 0.44 (0.17-1.16) | 0.097 |  |
| Concentric hypertrophy | 2 (6.3) | 81 (44.0) | 0.31 (0.07-1.39) | 0.125 |  |
| **Cardiovascular death** |  |  |  |  | 0.355 |
| Normal geometry | 7 (3.5) | 87 (13.9) | 1.03 (0.45-2.37) | 0.948 |  |
| Eccentric hypertrophy | 2 (4.2) | 70 (23.3) | 0.56 (0.13-2.43) | 0.438 |  |
| Concentric remodeling | 1 (1.4) | 50 (22.4) | 0.16 (0.02-1.20) | 0.075 |  |
| Concentric hypertrophy | 1 (3.1) | 43 (23.4) | 0.28 (0.03-2.26) | 0.232 |  |
| **Thromboembolism** |  |  |  |  | 0.762 |
| Normal geometry | 13 (6.4) | 66 (10.5) | 1.34 (0.70-2.60) | 0.378 |  |
| Eccentric hypertrophy | 3 (6.3) | 34 (11.3) | 0.93 (0.26-3.33) | 0.908 |  |
| Concentric remodeling | 3 (4.1) | 30 (13.5) | 0.27 (0.08-0.95) | 0.042 |  |
| Concentric hypertrophy | 5 (15.6) | 29 (15.8) | 1.96 (0.56-6.86) | 0.293 |  |
| **Major bleeding** |  |  |  |  |  |
| Normal geometry | 5 (2.5) | 26 (4.1) | 0.98 (0.35-2.76) | 0.972 |  |
| Eccentric hypertrophy | 1 (2.1) | 12 (4.0) | 0.29 (0.03-2.90) | 0.293 |  |
| Concentric remodeling | 2 (2.7) | 9 (4.0) | 0.46 (0.07-3.17) | 0.431 |  |
| Concentric hypertrophy | 0 | 4 (2.2) | - |  |  |

HR = hazard ratio; CI = confidence interval; RAASi = renin-angiotensin-aldosterone system inhibitors.
